# Antibody CD133 Biofunctionalization of Ammonium Acryloyldimethyltaurate and Vinylpyrrolidone Co-Polymer-Based Coating of the Vascular Implants

**DOI:** 10.3390/ma13245634

**Published:** 2020-12-10

**Authors:** Przemysław Sareło, Maciej Duda, Marlena Gąsior-Głogowska, Edyta Wysokińska, Wojciech Kałas, Halina Podbielska, Magdalena Wawrzyńska, Marta Kopaczyńska

**Affiliations:** 1 Department of Biomedical Engineering, Faculty of Fundamental Problems of Technology, Wrocław University of Science and Technology, Wybrzeże Stanisława Wyspiańskiego 27, 50-370 Wrocław, Poland; przemyslaw.sarelo@pwr.edu.pl (P.S.); maciej.duda@pwr.edu.pl (M.D.); marlena.gasior-glogowska@pwr.edu.pl (M.G.-G.); halina.podbielska@pwr.edu.pl (H.P.); 2 Department of Experimental Oncology, Ludwik Hirszfeld Institute of Immunology and Experimental Therapy, Polish Academy of Sciences, Rudolfa Weigla 12, 53-114 Wrocław, Poland; edyta.wysokinska@hirszfeld.pl (E.W.); wojciech.kalas@hirszfeld.pl (W.K.); 3 Department of Preclinical Studies, Faculty of Health Sciences, Wrocław Medical University, Kazimierza Bartla 5, 51-618 Wrocław, Poland; magdalena.wawrzynska@umed.wroc.pl

**Keywords:** bioresorbable polymer-based coating, vascular implants, antibody immobilization, stent surface biocompatibility

## Abstract

Current vascular stents, such as drug eluting stents (DES), have some serious drawbacks, like in stent restenosis and thrombosis. Therefore, other solutions are sought to overcome these post-implantations complications. These include the strategy of biofunctionalization of the stent surface with antibodies that facilitate adhesion of endothelial cells (ECs) or endothelial progenitor cells (EPCs). Rapid re-endothelialization of the surface minimizes the risk of possible complications. In this study, we proposed ammonium acryloyldimethyltaurate/vinylpyrrolidone co-polymer-based surface (AVC), which was mercaptosilanized in order to expose free thiol groups. The presence of free thiol groups allowed for the covalent attachment of CD133 antibodies by disulfide bridges formation between mercaptosilanized surface and cysteine of the protein molecule thiol groups. Various examinations were performed in order to validate the procedure, including attenuated total reflection–Fourier transform infrared spectroscopy (ATR-FTIR) and Fourier transform Raman spectroscopy (FT-Raman), atomic force microscopy (AFM) and scanning electron microscopy (SEM). By means of ATR-FTIR spectroscopy presence of the CD133 antibody within coating was confirmed. In vitro studies proved good biocompatibility for blood cells without induction of hemolytic response. Thus, proposed biofunctionalized CD133 antibody AVC surface has shown sufficient stability for adapting as cardiovascular implant coating and biocompatibility. According to conducted in vitro studies, the modified surface can be further tested for applications in various biological systems.

## 1. Introduction

Nowadays, numerous studies are carried out on coatings that are biofunctionalized with various biocompounds and biomolecules in order to obtain the coating specific properties. The functionalized material surfaces with antibodies are of particular interest. Immobilized anti-epithelial cell adhesion antibodies have found application in circulating tumor cells capture to prevent metastases development [1] in endothelial progenitor cells capture, which differentiate into a mature endothelium, thus increasing cardiovascular stents biocompatibility [2,3,4,5,6,7] in biosensor design and construction as well as diagnostic platforms with significantly improved detection efficiency [8,9]. Nevertheless, the critically important factor to be considered still remains, like immobilization efficiency of a well-oriented antibody onto the surface to achieve high molecule loading with affinity retention [9,10]. The different surface antibody immobilization methods have been extensively studied. Among various strategies, the physical adsorption [11,12], covalent bonding [11,12,13,14] or bio-affinity immobilization approach [15] can be distinguished.

The application of the endothelial cell (EC) and its progenitor cell specific (endothelial progenitor cell – EPC) antibodies have, therefore, found a special place in the surface modification of cardiovascular stents. Bare metal stents (BMS) were first introduced in the mid 1990s, quickly becoming a very powerful tool in modern interventional cardiology. This approach allowed to overcome some of the major difficulties of the early balloon angioplasty procedure, such as an elastic recoil and blood vessel reocclusion in the early stage of the intervention. However, some of the long-term problems, like restenosis and thrombosis still remained unsolved [16]. To overcome some of these problems, subsequently, drug eluting stents (DES) were developed. First generation of DES was introduced in the early 2000s and consisted of three main components: Metal scaffold, polymer coating and drug incorporated into the polymer structure [17,18]. The next step in the intravascular implants improvement were the bioresorbable scaffolds (BRS). Their primary objectives were to provide a temporary vessel support, prevent acute and late recoil, avoid stent fracture and also to address the issue of chronic inflammation and late stent thrombosis [19,20,21].

Apart from stent structure and material modifications, there are also specific implant surface biofunctionalizations proposed, including various biomolecules and bionanocomposites. Some of these methods use polymeric nanoparticles architecture wherein drug molecules are encapsulated and delivered to a target of interest [22]. Another approach to stents biofunctionalization is to cover its surface with bionanocomposite with immobilized biomolecules designed to capture endothelial progenitor cells [2,23]. Once captured EPCs differentiate into a mature endothelium, thus increasing stents biocompatibility [4,5,7,24]. One of the approaches was to use immobilization of anti-CD34 antibodies, for biofunctionalization of stent, but such surface tends to have insufficient biocompatibility. Therefore, attracting EPCs emerged as alternative strategy. Use of EPCs specific anti-CD133 coated surface has been shown to have high reendothelialization rate, when used in stents [23,24].

In this work, we present novel coating facilitating anti-CD133 antibodies immobilization. Biomolecules were immobilized on a surface covered by the thin film prepared with ammonium acryloyldimethyltaurate/vinylpyrrolidone co-polymer (AVC). AVC belongs to the group of AMPS^®^ polymers, whose chemical structure is based on acrylamido-2,2-dimethylpropanesulfonic acid. The co-monomer in AVC is vinylpyrrolidone. This co-polymer has a high degree of cross-linking despite the repulsion associated with negatively charged sulfonic acid residues in the one of the co-monomers. Nevertheless, the negatively charged sulfonic acid groups are responsible for strong interactions with polar solvents, leading to the swelling of the network structure and to the higher overall viscosity. AVC polymers with a high degree of cross-linking have a more flexible network, resulting in higher plasticity values and a more flexible characteristic [25]. These properties can be a great advantage in the context of expansion stent strut, which can prevent the biofunctionalized coating from cracking.

In our study, the biocompatibility of the coating was examined. The potential of inducing hemolysis of erythrocytes as an important part of hemocompatibility assessment, crucial for vascular implants was performed. The biocompatibility screening tests the anchoring type cell line was chosen for adhesion assessment. It is essential to obtain a coating that will not significantly interact with the cells of the immune system, thereby initiating a series of complications related to the implantation of the biomaterial.

**Figure 1:**
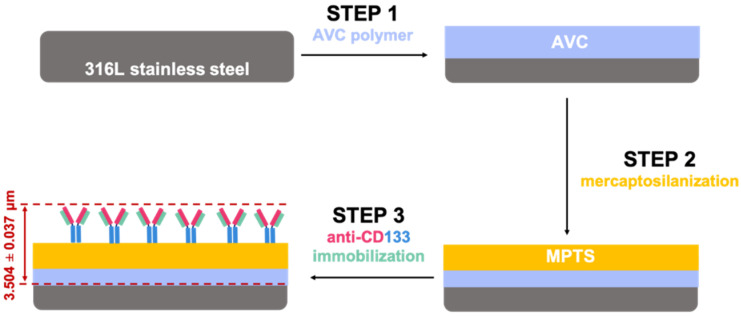
The general concept of the work—a schematic representation of the proposed method of antibody immobilization on the ammonium acryloyldimethyltaurate/vinylpyrrolidone co-polymer-based coating surface.

In this study we propose a complete procedure to successfully functionalize the surface of the material used in the production of cardiovascular stents—316 L stainless steel (Figure 1). The evaluation of a newly prepared biofunctional stent coating was carried out by atomic force microscopy (AFM), scanning electron microscopy (SEM), attenuated total reflection–Fourier transform infrared spectroscopy (ATR-FTIR), Fourier transform Raman spectroscopy (FT-Raman) and confocal microscopy to determine the impact of specific functionalization on distribution, nanostructural characteristics and intramolecular interactions of anti-CD133 molecules as well as their neutral interaction with macrophages.

## 2. Materials and Methods

### 2.1. AVC Film Formation

**Figure 2:**
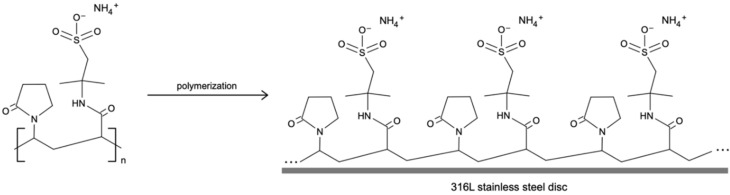
AVC co-polymer chemical structure. Polymerization reaction by drying at the room temperature.

Initially, 316 L stainless steel discs (Balton, Warsaw, Poland), 10 mm in diameter, were selected as the base material for the coating. Firstly, discs were covered with ammonium acryloyldimethyltaurate/vinylpyrrolidone (Aristoflex^®^ AVC) co-polymer (Clariant International Ltd., Muttenz, Switzerland) solution (0.1% in deionized water m/v). On the center of each discs approximately 100 µL solution was deposited. Then, discs were placed in TC100 spin coater (MTI Corporation, Richmond, CA, USA), spun at 3000 rpm for 5 s and left to dry in the air at room temperature. The loss of water during drying made polymerization reaction possible followed by the thin film formation. Schematic reaction is shown in Figure 2. A newly prepared AVC coating were stored in sealed plastic Petri dishes (TPP Techno Plastic Products AG, Trasadingen, Switzerland) at 4 °C for the next step, which was mercaptosilanization.

### 2.2. Mercaptosilanization

**Figure 3:**
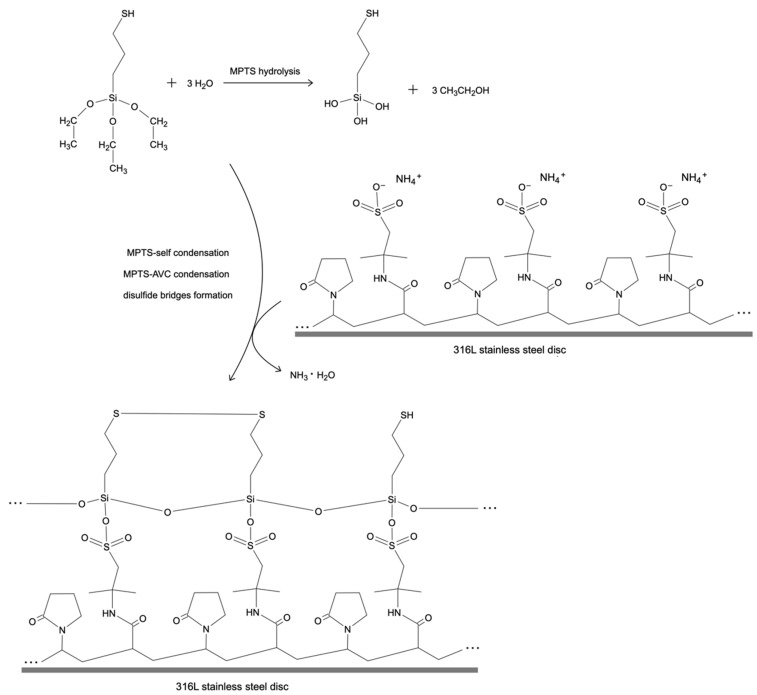
The schematic representation of chemical reactions which took place within the mercaptosilanization step of coating formation.

For the mercaptosilanization step, an ethanolic (Avantor Performance Materials, Gliwice, Poland) solution of (3-mercaptopropyl)triethoxysilane (MPTS) (Sigma-Aldrich, St. Louis, MO, USA) at concentration 9% *v*/*v* and Triton X-100 (Sigma-Aldrich, St. Louis, MO, USA) at concentration 0.5% *v*/*v*, was prepared. Discs covered with AVC-based film were dipped in the MPTS solution. Then, the discs were stored in the heater (CLN 15 STD, POL-EKO-APARATURA sp. j., Wodzisław Śląski, Poland) for 1 h at 60 °C and subsequently washed with ethanol and dried in air at the room temperature. A schematic representation of chemical reactions that took place (e.g., MPTS hydrolysis, MPTS-self condensation, MPTS-AVC condensation and side formation of disulfide bridges) are shown in Figure 3.

### 2.3. Surface Biofunctionalization with Anti-CD133 Antibodies

The anti-CD133 immobilization was conducted by means of disulfide bridges formation between antibody cysteine thiol groups and thiol groups introduced within mercaptosilanization step. To ensure that mercaptosilanized discs’ surface is covered with free thiol groups, 10 mM DTT (Thermo Fisher Scientific, Waltham, MA, USA) reduction reaction was performed. In order to break the possibly formed disulfide bonds on discs covered with coating, DTT solution was deposited. Discs with DTT were incubated for 1 h at 37 °C, then rinsed with phosphate buffered saline (PBS) (Sigma-Aldrich, St. Louis, MO, USA) and deionized water. Afterwards, they were dried in air at room temperature. The anti-CD133 immobilization process was carried out by depositing 20 µL of 5 µg/mL monoclonal CD133-antibody (Cloud-Clone Corp., Katy, TX, USA) in 10 mM PBS solution onto a disc with newly prepared coating and thermal treatment in a heater for 1 h at 37 °C. After incubation, discs were washed with PBS and left to dry at room temperature. Samples were stored in sealed plastic Petri dishes at 4 °C for examinations.

### 2.4. ATR-FTIR Spectroscopy

ATR-FTIR spectra were recorded using a Nicolet 6700 FT-IR Spectrometer (Thermo Fisher Scientific, Waltham, MA, USA) with Golden Gate Mk II ATR Accessory with Heated Diamond Top-plate (PIKE Technologies, Fitchburg, WI, USA). The spectrometer was continuously purged with dry air. All spectra were collected in the range of 4000–400 cm^−1^ with a spectral resolution of 4 cm^−1^, averaging 128 scans. Directly before sampling, the background spectrum of diamond/air was recorded as a reference (512 scans, 4 cm^−1^). All spectra were registered at temperature of 37 °C (98.6 °F). All spectra were analyzed using the OriginPro (ver. 2019, OriginLab Corporation, Northampton, MA, USA).

### 2.5. FT-Raman Spectroscopy

FT-Raman spectra were obtained with a Nicolet NXR 9650 FT-Raman spectrometer (Thermo Fisher Scientific, Waltham, MA, USA) equipped with Nd:YAG excitation laser (1064 nm, 250 mW), InGaAs detector and MicroStage extension. All samples spectra were collected in the range of 4000–250 cm^−1^ with a spectral resolution of 4 cm^−1^, averaging 128 scans. All spectra were analyzed using the OriginPro (ver. 2019, OriginLab Corporation, Northampton, MA, USA).

### 2.6. Scanning Electron Microscopy

In order to obtain scanning electron microscope images of the 316 L stainless steel discs covered with AVC co-polymer functionalized with the CD133 antibody with or without mercaptosilanization and DTT reaction steps, samples were mounted on microscope stubs with carbon tape, sputtered with carbon using auto carbon coater JOEL JEC-530 (JOEL Ltd., Tokyo, Japan) and observed by means of scanning electron microscope JOEL JSM661OLV (JOEL Ltd., Tokyo, Japan) at 15 kV of a beam voltage.

### 2.7. Atomic Force Microscopy

In order to determine the presence of the immobilized anti-CD133, atomic force microscopy measurements were performed. The same coating was prepared on the freshly air-cleaved muscovite-mica square discs (Labnatek, Warsaw, Poland), size approximately 10 mm by 10 mm. Images were acquired in the tapping mode by means of Nanoscope IIIa scanning probe microscope with Extender Module (Brucker, Billerica, MA, USA) in an air atmosphere at room temperature. The sample was adjusted with the optical light microscope (Nanoscope Optical Viewing System, Brucker, Billerica, MA, USA). Etched silicon cantilevers (Olympus, Shinjuku, Tokyo, Japan) were used with typical resonance frequency in the range of 200–400 kHz, a spring constant of 42 N/m and the tip diameter 10 nm. The set value of the probes’ vibration amplitude was maintained, by the feedback system, up to 80% of the free oscillation amplitude of the probe. The scanning frequency was between 0.500–1.500 Hz and the scanning angle was set to 0°. AFM images were processed in the Nanoscope v.6.13 software (Veeco Instruments Inc., Plainview, NY, USA). By the default software function the image noise was reduced, the plane of the images was leveled.

### 2.8. Cell Cultures

RAW264.7 macrophage cell line was maintained in high-glucose Dulbecco’s Modified Eagle’s Medium (DMEM; IITD, Wroclaw, Poland) supplemented with 10% fetal calf serum (FCS, GibcoTM, Thermo Fisher Scientific, Waltham, MA, USA), glutamine (Sigma-Aldrich, St. Louis, MO, USA), HEPES (Sigma-Aldrich, St. Louis, MO, USA), sodium pyruvate (Sigma-Aldrich, St. Louis, MO, USA) and Antibiotic and Antimycotic Solution (Sigma-Aldrich, St. Louis, MO, USA).

### 2.9. Hemolysis Assay

Analogous coatings have been deposited on the mica discs. Such prepared mica discs were used in the in vitro experiments. Then, the surfaces prepared on a mica discs were placed in the well of 24-well plate (Nunc, Thermo Fisher Scientific, Waltham, MA, USA). The blood samples from healthy volunteers were collected into BD Vacutainer Plastic Blood Collection Tubes (Becton, Dickinson and Company, Franklin Lakes, NJ, USA) with K2EDTA and centrifuged at 500× *g* for 5 min. The blood plasma was discarded, and hematocrit was washed two times with 150 mM NaCl (IITD, Wrocław, Poland) and with PBS pH 7.4 (IITD, Wrocław, Poland) at 500× *g* for 5 min. The final pellet was diluted 1:49 (*v*/*v*) in PBS pH 7.4 and 0.5 mL of diluted erythrocytes was added onto discs and incubated 1 h at 37 °C. Next, the discs were removed, and plate was centrifuged at 500× *g* for 5 min. Triplicates (100 µL) of supernatant were transferred to 96-well plate (Nunc, Thermo Fisher Scientific, Waltham, MA, USA) and 450 nm absorbance at corresponding to free hemoglobin was measured with a VallacVictor2 plate reader (PerkinElmer, Waltham, MA, USA). For positive control erythrocytes were lysed with 1% Triton X-100 (Sigma-Aldrich, St. Louis, MO, USA) in PBS pH 7.4 and read out was regarded as 100% of hemolysis. Control erythrocytes were incubated in PBS pH 7.4. The local ethics committee at the Institute of Immunology and Experimental Therapy, Polish Academy of Sciences, Wrocław Poland, approved the study (permission # 64/2015 and 79/2015).

### 2.10. Bright-Field Microscopy

The surfaces prepared on a mica discs were placed in the plate of 96-well (Nunc, Thermo Fisher Scientific, Waltham, MA, USA). On prepared plate 10^4^ RAW264.7 cells per well were added. After 48 h incubation on the disc’s cells were fixed with 4% (w/w) formaldehyde in PBS (20 min) and washed thrice with PBS. To prevent probe drying 100 µl of PBS were added to each well. The Leica TCS-SPE confocal microscope (Leica Microsystems, Wetzlar, Germany) connected with the LAS AF microscope software (Leica Microsystems, Wetzlar, Germany) was used to visualize the macrophages placed on the functionalized surface of mica discs. Images were acquired in the transmission mode.

### 2.11. Cell Counting

The surfaces prepared on a mica discs were placed in the well of 96-well plate (Nunc, Thermo Fisher Scientific, Waltham, MA, USA). Then, the 10^4^ RAW264.7 cells per well were added. After 48 h incubation macrophages were collected by trypsinization (Trypsine/EDTA, IITD, Wrocław, Poland) and counted using MOXI Z Mini Automated Cell Counter (Orflo Technologies, Ketchum, ID, USA).

## 3. Results

### 3.1. Spectroscopic Evaluation of Functionalized Surfaces

**Figure 4:**
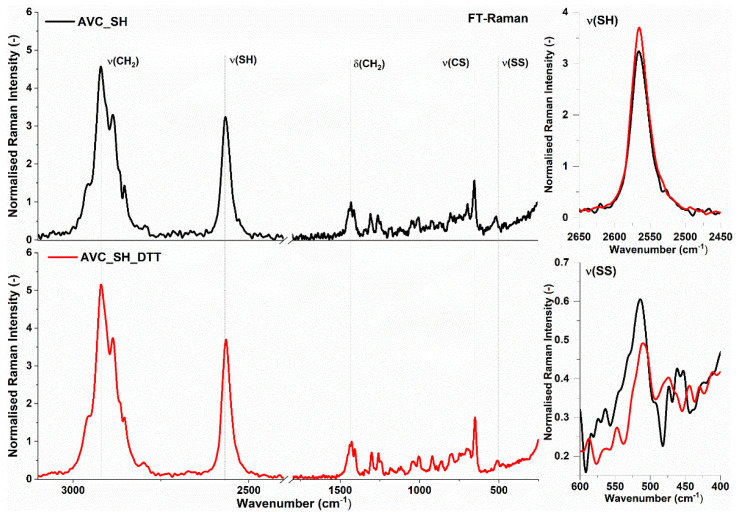
FT-Raman spectra of the ammonium acryloyldimethyltaurate/vinylpyrrolidone co-polymer (AVC) after mercaptosilanization (**upper**) and incubation with dithiothreitol (DTT) at 37 °C (98 °F) for 1 h (**bottom**) with spectra in the SH and SS stretching vibrations ranges (**right**).

Free thiol groups are required to bind the CD133 antibody to the mercaptosilanized AVC-coated surface. Dithiothreitol (DTT) was used to break possible disulfide bonds created after the mercaptosilanization step. This process was monitored by means of FT-Raman spectroscopy. The result of a treatment AVC with DTT is an increasing amount of free thiol groups (Figure 4) [26]. The band at 515 cm^−1^ arising from the S-S stretching vibrational mode of a disulfide bond lower its intensity. In turn, for the band at 2565 cm^−1^ assigned to sulfhydryl (S-H) groups an increment is visible, as well as ratio of νSH/δCH bands. The value of the ratio of νSH/δCH is equal to 3.2 and 3.7, before and after the mercaptosilanization step, respectively. The amount of free thiol groups increased by approximately 15% due to DTT treatment.

**Figure 5:**
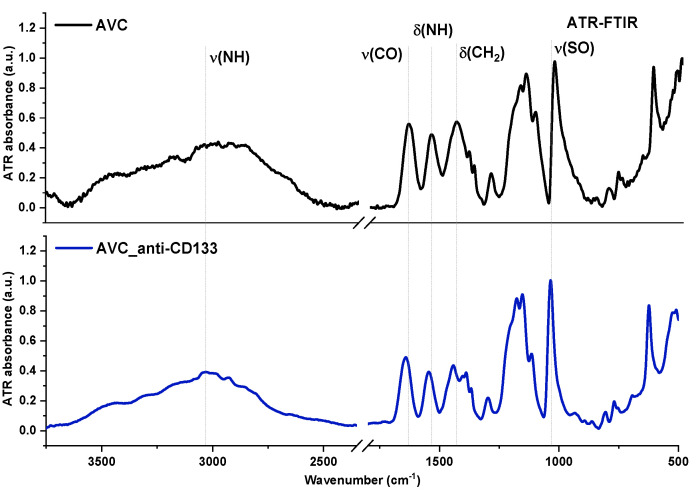
Attenuated total reflection–Fourier transform infrared spectroscopy (ATR-FTIR) spectra of the AVC (**upper**) and AVC with deposited solution of anti-CD133 (**bottom**).

**Figure 6:**
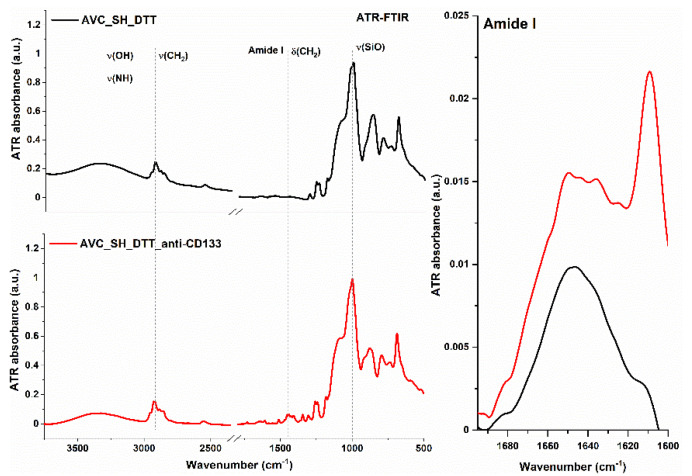
ATR-FTIR spectra of the AVC after mercaptosilanization and reduction before (**upper**) and after (**bottom**) deposited solution of anti-CD133 with the amide I range enlarged (**right**).

Figure 5 presents an ATR-FTIR spectrum obtained from the 316 L stainless steel discs coated with AVC co-polymer before (AVC) and after adding of monoclonal CD133-antibody solution (AVC_anti-CD133). The insignificant influence on the spectral signature was observed, which allows the assumption that antibody molecules did not bind to the surface. In turn, after mercaptosilanization, the antibody is able to attach to the surface, which is confirmed by the presence of amide bands, which are characteristic for peptide bonds, in infrared spectrum (AVC_SH_DTT_anti-CD133) as shown in Figure 6. Although ATR-FTIR spectrum was dominated by Si-O absorption bands at approximately 1000 cm^−1^, the amide I occurred in the range of 1710–1590 cm^−1^. Maximum of the amide I, corresponding mainly to C=O stretching vibrations of peptide bonds, observed around 1610 cm^−1^ was typical for β-sheet structures. The band at 1650 cm^−1^ can be assigned to globular fragments [27]. In ATR-FTIR spectrum of AVC without antibody (AVC_SH_DTT) in range of 1700–1600 cm^−1^, only a single band origin from stretching vibrations of C=O groups was visible instead of complex amide I band.

### 3.2. Nanostructural Characterization of Biofunctionalized Surfaces

**Figure 7:**
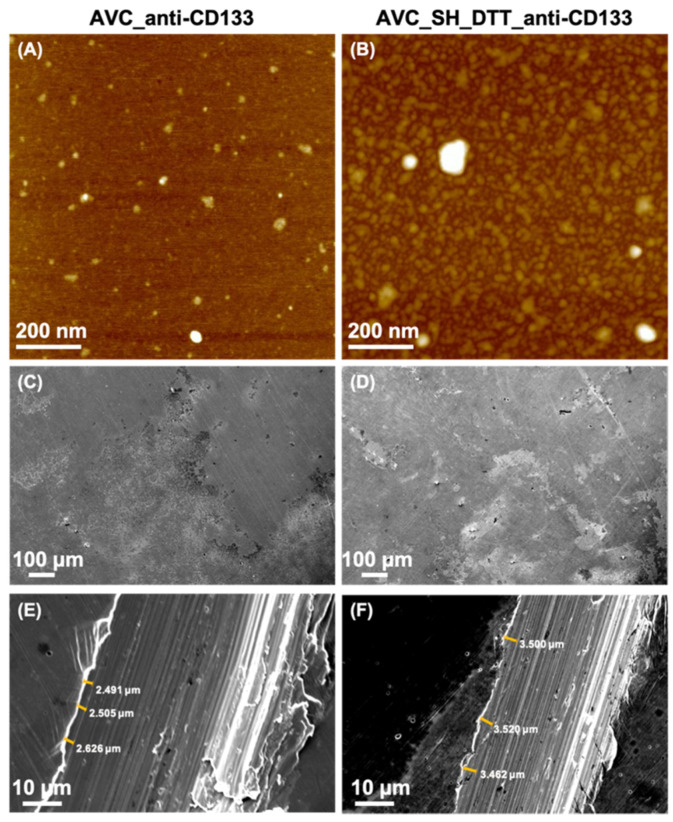
Atomic force microscopy (AFM) images of the AVC with deposited solution of anti-CD133 and AVC after mercaptosilanization, reduction and anti-CD133 immobilization (**A**,**B**, respectively). SEM images of the biofunctionalized surfaces (**C**–**F**) with the thickness of the coating measurement marked (**E**,**F**).

The nanostructural characterization of functionalized surface AVC with deposited solution of CD133 antibody (AVC_anti-CD133) and AVC after mercaptosilanization, reduction and anti-CD133 immobilization by disulfide bridges formation (AVC_SH_DTT_anti-CD133) was performed by means of AFM and SEM. AFM topography imaging confirmed that in both cases, with or without mercaptosilanization and reduction step, the coating is rather smooth, even in the nanoscale (Figure 7A,B). Nevertheless, introduction of the additional steps in the coating preparation (i.e., mercaptosilanization and reduction) provides higher degree of antibody immobilization on the surface. Moreover, the particle distribution is in this case also homogeneous within the entire analyzed surface. The lack of the above-mentioned steps in the synthesis of the coating results in well dispersed particles distribution, with their lower degree of bonding, what is a result of adsorption antibody on the surface rather than formation of more permanent bond to the polymer-based layer (i.e., disulfide bridges). SEM imaging confirmed that there are not visible surface discontinuities present, neither in AVC_anti-CD133 (Figure 7C) nor in AVC_SH_DTT_anti-CD133 (Figure 7D). The average thickness of AVC_anti-CD133 and AVC_SH_DTT_anti-CD133 coating is 2.541 ± 0.074 μm and 3.504 ± 0.037 μm, respectively (Figure 7E,F).

### 3.3. Biosafety Assessment of Biofunctionalized AVC Surfaces

**Figure 8:**
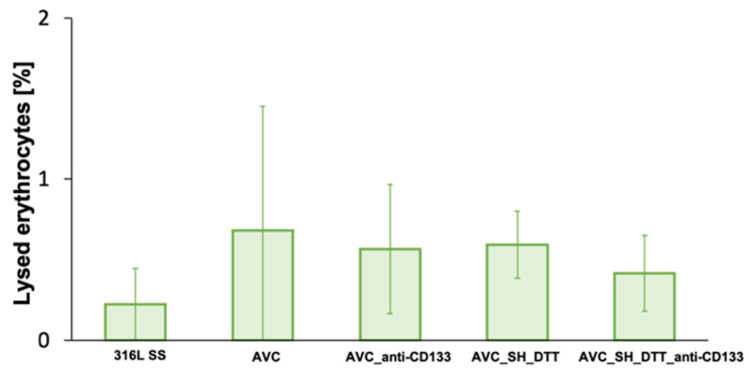
Hemolysis assay results. Erythrocytes lysed with 1% Triton X-100 were regarded as a 100% of hemolysis.

Surface-modified and unmodified mica discs were examined for their hemolytic potential upon contact with human blood. The erythrocytes were incubated with AVC, AVC_anti-CD133, AVC_SH_DTT and AVC_SH_DTT_anti-CD133 modified discs or unmodified discs for 1 h at the temperature of 37 °C. Percentages of lysed erythrocytes not exceeding 5%, indicates absence of hemolytic property of tested mica discs (Figure 8). Presented results indicate lack of hemolytic activity of tested surfaces. AVC-based surfaces were already regarded as biocompatible [25]. We show that AVC polymer surface biofunctionalized with anti-CD133 did not exhibit the toxicological activity against erythrocytes or cultured cells.

**Figure 9:**
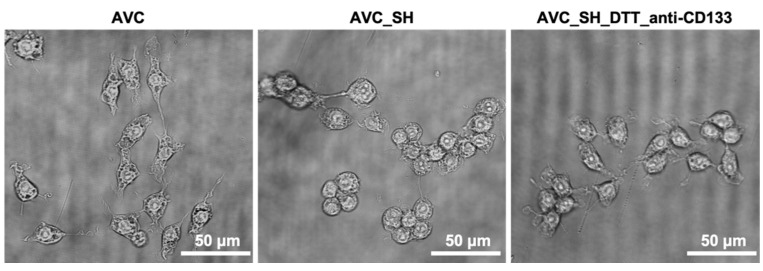
Bright-field microscopy of the macrophages on the functionalized surface with AVC, with AVC after mercaptosilanization and with AVC after mercaptosilanization, reduction and anti-CD133 immobilization.

**Figure 10:**
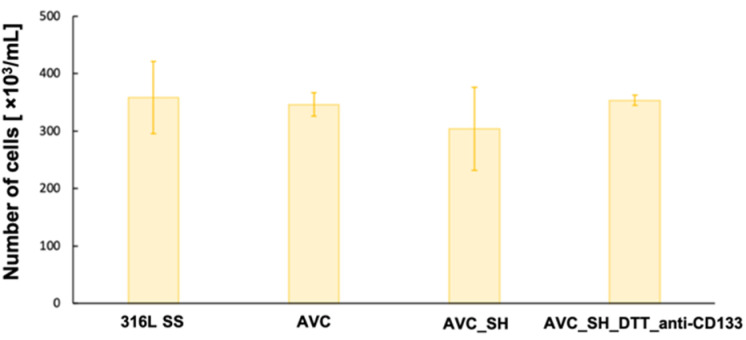
Macrophages concentration after 48 h incubation on the functionalized surface with AVC, with AVC after mercaptosilanization and with AVC after mercaptosilanization, reduction and anti-CD133 immobilization.

To confirm basic compatibility of newly developed AVC-based surfaces, adherent RAW264.7 cells were incubated on these surfaces. First, the cells were able to attach and grow on all tested surfaces. Secondly, the morphology of the cells that were grown for 48 h on AVC surfaces was not affected, especially in the case of AVC and CD133 antibody dotted AVC surfaces (Figure 9). Moreover, the number of viable cells from tested surfaces was similar to control (cell culture plasticware) indicating that coating with AVC-based material does not affect growth of cells (Figure 10). Reassuming, in vitro studies confirm that AVC-based surfaces, especially CD133 antibody doped surface, are not hemotoxic and are suitable for further in vivo studies and can be successfully tested for applications in biological systems.

## 4. Discussion

In this study, we have reported a multistep protocol of biofunctionalization of the 316 L stainless steel discs surface by means of preparation ammonium acryloyldimethyltaurate/vinylpyrrolidone co-polymer-based thin film, introduction of the (3-mercaptopropyl)triethoxysilane reagent to obtain exposed free thiols groups on the material surface—potentially formatted disulfide bridges were broken by reaction with dithiothreitol—and covalent binding CD133 antibody by means of disulfide bridges formation between surface exposed free thiols groups with the cysteine free thiol groups within antibody molecule. The presence of the free thiol groups on the material surface was verified by FT-Raman spectroscopy, where a higher intensity of SH stretching mode was observed in comparison to plain AVC film. Moreover, ATR-FTIR spectroscopy revealed the presence of the CD133 antibody molecules on the surface, what was confirmed by amide I band. Low intensity of the band was a result of low concentration of the antibody used in the experiment. Nevertheless, its concentration was sufficient to be characterized by the exploited technique. The nanostructural analysis of coatings provided important information in the context of the homogenous distribution of biomolecules within the coating, and, additionally, proved that the coating has no evident surface discontinuities signs. Tested functionalized surfaces exhibit good biocompatibility for blood cells. Prepared coatings did not induce any hemolytic response. Discs did not affect the cells morphology. However, it is also vitally important that functionalized discs had no impact on macrophages cells attachment and grow. In vitro studies confirmed that anti-CD133 biofunctionalized polymer-based surface is biocompatible and suitable to further in vivo studies and can be tested for applications in biological systems.

## Abbreviations

AFM	Atomic Force Microscopy
ATR-FTIR	Attenuated Total Reflection Fourier Transform Infrared Spectroscopy
AVC	Ammonium Acryloyldimethyltaurate/Vinylpyrrolidone co-polymer
BMS	Bare Metal Stents
BRS	Bioresorbable Scaffolds
DES	Drug Eluting Stents
DTT	Dithiothreitol
ECs	Endothelial Cells
EPCs	Endothelial Progenitor Cells
FCS	Fetal Calf Serum
FT-Raman	Fourier Transform Raman Spectroscopy
MPST	(3-Mercaptopropyl) trimethoxysilane
PBS	Phosphate Buffered Saline
SEM	Scanning Electron Microscopy
